# Group Outdoor Health Walks Using Activity Trackers: Measurement and Implementation Insight from a Mixed Methods Feasibility Study

**DOI:** 10.3390/ijerph17072515

**Published:** 2020-04-07

**Authors:** Katherine N. Irvine, Melissa R. Marselle, Alan Melrose, Sara L. Warber

**Affiliations:** 1 Social, Economic and Geographical Sciences, James Hutton Institute, Craigiebuckler, Aberdeen AB14 8QH, UK; 2 Helmholtz Center for Environmental Research-UFZ, Department of Ecosystem Services, Permoserstr 15, 04318 Leipzig, Germany; melissa.marselle@idiv.de; 3 German Centre for Integrative Biodiversity Research (iDiv) Halle-Jena-Leipzig, Deutscher Platz 5e 04103 Leipzig, Germany; 4 Alan Melrose Consultancy Ltd., 1 Balnastraid Cottages, Dinnet, Aboyne AB34 5NE, UK; alanmelrose57@gmail.com; 5 Department of Family Medicine, University of Michigan Medical School, 1018 Fuller St, Ann Arbor, MI 48104-1213, USA; swarber@umich.edu; 6 European Centre for Environment and Human Health, University of Exeter Medical School, Truro TR1 3HD, UK

**Keywords:** biopsychosocial–spiritual health, green exercise, health promotion, implementation research, nature-based interventions, nature-based therapies, nearby nature, older adults, physical activity, wellbeing

## Abstract

Outdoor walking groups are nature-based interventions (NBIs) that promote health and wellbeing by modifying individual behaviour. The challenges of such NBIs include the motivation of inactive adults to participate and measurement issues. This feasibility study investigates a 12-week group outdoor health walk (GOHW) incorporating activity trackers and use of a holistic health and wellbeing measure, the Self-sasessment of Change (SAC) scale. A mixed methods design explored participant recruitment and retention, programme delivery, and measures of physical activity and health and wellbeing. Walker data included: pre-post questionnaires, daily step counts, and interviews. Programme delivery information included: weekly checklists, staff reflections, stakeholder meeting minutes, and a report. Thirteen adults (age 63–81, 76% female) joined and completed the activity tracker GOHW. Activity trackers motivated walkers to join and be more active but complicated programme delivery. Activity trackers allowed the quantification of physical activity and the SAC health and wellbeing measure was easy to use. By week 12, all participants met national physical activity guidelines. Clinically relevant changes on the SAC scale included: sleeping well, experiencing vibrant senses, and feeling energised, focused, joyful, calm and whole. Results illustrate the feasibility of using activity trackers to motivate engagement in and provide a measure of physical activity from GOHWs. The SAC scale offers a promising measure for nature–health research. A conceptual model is provided for the development of future large-scale studies of NBIs, such as group outdoor health walks.

## 1. Introduction

Physical inactivity has recognised consequences for individual health, public health [1] and associated implications for healthcare services and expenditure [2,3]. It has, for example, been estimated that, in Scotland, “inactivity contributes to nearly 2500 deaths and costs the NHS [National Health Service] around £91 million per year” [4]. Addressing the problems of physical inactivity, along with associated physical and other (e.g., mental) health consequences, are global priorities [5]. Health promotion interventions are essential in order to reduce healthcare demands and treatment costs; ideally these interventions should be less medicalised, more person-centred and widely available, as well as effective and cost-effective [6]. The importance of partnerships across sectors, community-level involvement, and the need for evaluation has also been recognised to meet these challenges [7].

Walking is a widely endorsed way to increase physical activity [5,8]. Walking is an accessible, low risk and inexpensive form of physical activity [8] that can prevent and treat non-communicable diseases [5], such as cardiovascular disease [9] and obesity [10,11], and improve mental health [12]. Whilst walking is the most common form of physical activity in the US and the UK [8,13,14], only half of adults in the US [15] and two-thirds of adults in the UK [16] meet the recommended levels of physical activity. Within Scotland there has been little change over time in those classed as ‘inactive’, with older adults least likely to meet physical activity guidelines [4]. Finding ways to increase the uptake of walking in those who are physically inactive could contribute to meeting physical activity guidelines and reducing health risks. Physical and social environments both influence one’s decision to walk; whilst changing the built environment is costly, changing the social environment, i.e. walking with others, may provide a cost-effective way of encouraging people to walk [17]. Individuals are more likely to walk when with another person [18] and group walking has been found to increase physical activity [19] as well as improve adherence [20]. However, studies of interventions that use the social environment to promote walking are lacking [21]. 

### 1.1. Rationale for Group Walks in Nature

Outdoor exercise groups are nature-based interventions (NBIs), usually walking, that promote wellbeing or prevent chronic health conditions by modifying individual behaviour [22]. With growing interest in the health promoting effects of the natural environment [23,24,25] and the increasing availability of ‘walking for health’ programmes, one potential health solution for physical inactivity is group walks in nearby nature. 

Systematic reviews and meta-analyses demonstrate that walking in nature has added health benefits over walking in urban spaces [26] or indoors [26,27]. Hanson and Jones [28] conclude in their systematic review “that outdoor walking groups have health benefits over and above making people increase physical activity” (p. 5). Indeed, group walks in nature have been shown to improve physical activity [29] as well as mental health and wellbeing [30,31,32,33,34]. In a large-scale evaluative study, Marselle et al. [33] found that group walks in natural environments were associated with better mental health. Additional analyses found that group walks in farmland or green corridors were associated with a significant reduction in stress and negative emotions [35] and that elements associated with an outdoor group walk (e.g., biodiversity, naturalness, intensity, duration) affect wellbeing [36] and are mediated by the perceived restorativeness of the environment [37]. However, a major limitation of these previous works is the lack of pre-post health and wellbeing evaluations [29,33].

Group outdoor health walks (GOHWs) are an NBI that seeks to modify individual behaviour through exercise within a group in a natural environment [22]. GOHWs are defined as a “short, safe, social, local, low level, led walk” [38] that lasts for no more than an hour. These non-health service interventions are typically run by third-sector or community agencies with locally based, trained volunteer walk leaders. A target audience for such walks are individuals who are relatively inactive and would benefit from being more physically active. 

GOHWs have been identified as providing a socially supportive setting that attracts people to commence and maintain participation [39,40], and are advocated as part of community-wide approaches to promote walking [8]. However, despite considerable research into why people use available areas of nature [41], a major issue remains unresolved: how to get people out to utilise nearby nature for their health and wellbeing. 

### 1.2. Activity Tracker—Motivator and Intermediate Measure

Advanced fitness or activity trackers are a rising trend in personal health technology [42] and their availability might attract people to join a GOHW. These trackers comprise multi-directional accelerometers along with software that can be accessed via a smartphone or a computer. The software is critical for providing an interpretation of the tracker data and supportive feedback to the wearer [43].

These devices can measure activity-related parameters, such as step counts, and may have the potential to promote increased physical activity and improved downstream health. A systematic review of randomised controlled trials (RCTs) [43] assessed whether activity trackers were effective in these two aspects of health promotion. Passive self-monitoring with or without goal setting was found to be ineffective for increasing physical activity, whereas the use of an activity tracker with goal setting was effective for increasing physical activity and weight loss. However, few studies assessed longer term, downstream health outcomes, such as quality of life [43]. In those that did, neither activity trackers alone nor integrative interventions were effective for these longer-term health outcomes. While the use of activity trackers remains under-evaluated [21], this systematic review suggests that activity trackers could be beneficial for increasing physical activity.

### 1.3. Measuring Downstream Health and Wellbeing

One of the challenges in determining the effects on longer term health outcomes may be the measures that are available. The World Health Organization defines health in its broader sense as “a state of complete physical, mental, and social wellbeing and not merely the absence of disease or infirmity” [44]. In the nature–health literature, multiple measures of health and wellbeing have been used. A recent review found that wellbeing measures tend to emphasise mental wellbeing [45]. However, Irvine and Warber [46] and Irvine et al. [41] have mapped the benefits of time spent in nearby nature onto a holistic biopsychosocial–spiritual model of health. This model encompasses the physical, cognitive, emotional, social, and spiritual dimensions of health and wellbeing [47,48,49,50].

Few existing measures contain all these dimensions in a single scale [51,52,53] and one of these has 317 items [53], presenting undue participant burden. The field of integrative medicine has faced this challenge and developed the Self-Assessment of Change (SAC) scale, a holistic measure of biopsychosocial–spiritual health. The SAC scale is sensitive to changes experienced by people engaged in complex interventions to promote health and wellbeing [54,55,56], thus it could be applicable for assessment of the biopsychosocial–spiritual health outcomes in NBI studies.

### 1.4. Feasibility Studies

Feasibility studies can provide a useful way to assess the acceptability of the processes associated with research and/or the development of new or modified interventions that promote health through behaviour change. The UK National Institute of Health Research (NIHR) describes these as ‘research done before a main study in order to answer the question ‘Can this study be done’ [57] (p. 2). Some relevant considerations include:

*recruitment capability and resulting sample characteristics, data collection procedures and outcome measures, acceptability and suitability of the intervention, the ability to manage and implement the intervention, and preliminary evaluation of participant responses to intervention*.

[58] (p. 1)

Such insight, often derived from designs that include qualitative methods alongside descriptive statistics, can be critical for determining the feasibility of larger scale studies, and hence inform the wise use of resources [59]. With increasing calls for evaluative research, such as RCTs or other top-tier research designs, to ‘identify drivers influencing the effectiveness of NBIs in enhancing health and wellbeing’ [22] (p. 2), it is important to examine such feasibility issues in relation to NBIs. 

### 1.5. Study Focus

This paper reports the findings from a transdisciplinary feasibility study of an NBI to increase physical activity and health benefits for older adults. The intervention introduced the use of advanced activity tracker technology into a GOHW. The programme organisers hoped to encourage health professionals to recommend the walking group and to motivate participants to join up and remain engaged. The study objectives were to:

1. Explore the contribution of activity trackers for the recruitment and retention of participants;
2. Understand the impact of activity tracker technology on programme implementation;
3. Field-test the use of step counts as a measure of physical activity and the SAC scale as a measure of biopsychosocial–spiritual health in studies of nature-based health promotion programmes.

## 2. Methods 

An observational pretest–posttest 12-week feasibility study was conducted using a convergent mixed methods design [60]. Mixed methods that include qualitative, quantitative and integrated data and analysis are particularly helpful in gleaning perspectives relevant to multiple stakeholders in feasibility studies [61,62]. These multiple sources of data can facilitate the triangulation and corroboration of insight [63]. The study was developed in consultation with the individuals involved in delivering a GOHW incorporating activity tracker technology and who, with researcher support, were responsible for participant recruitment, data collection, entry and anonymisation. Ethics was obtained from the James Hutton Institute’s Research Ethics Committee (52/2016). 

### 2.1. Nature-based Health Intervention—Activity Tracker GOHW

The activity tracker GOHW ran for 12 consecutive weeks (June–August 2016) and incorporated a wearable physical activity tracker (Storm Health, Edinburgh, UK) for each participant, an interactive online web platform (Living It Up), and individual goal setting. It was developed by a non-health third sector organisation (Active Cairngorms), that was already running outdoor walking groups, and delivered by a locally based trained volunteer Walk Leader. Activity trackers were synced weekly by the Walk Leader and progress was monitored through the use of automatically generated graphs. Graphs illustrated daily step counts over time and were used by the Walk Leader to provide feedback and facilitate goal setting for individual walkers. 

The activity tracker GOHW took place in the nearby nature in proximity to a village located in a national park in Scotland, UK. Covering approximately 4500 km^2^ (1740 square miles), with a population of 18,000 people (many of whom are 65 years old) [64], the national park is home to approximately 25% of the UK’s threatened plants and animal species and half of the park’s area is designated as of European importance for nature conservation [65]. Landscape types within the national park include forests, moorland, farmland, grassland, rivers and lochs as well as more manicured green spaces in the villages that are situated within the park. 

### 2.2. Recruitment

Walker recruitment was designed and conducted by the GOHW Programme Coordinator (hereafter referred to as the Walk Coordinator) and the local Walk Leader. Promotional materials were placed in local health centres and other community locations (e.g., notice boards) as well as distributed door-to-door. Direct outreach was made to medical doctors in the local health centre to encourage recommending the walks to appropriate individuals. These recruitment methods specifically sought to reach the inactive and those with health conditions who might benefit from increased physical activity (e.g., diabetes) who lived within the community in which the activity tracker GOHW was to take place.

### 2.3. Data Collection and Study Procedures 

**Figure 1:**
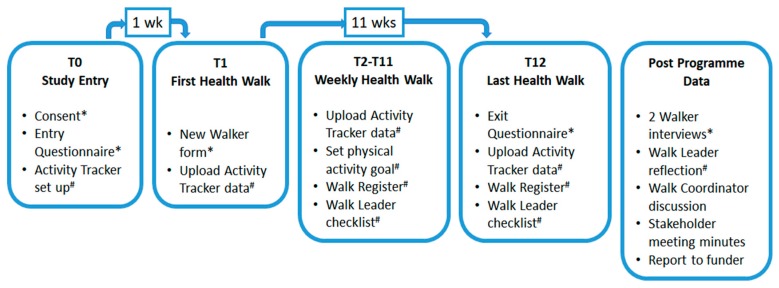
Data collection and study procedures for mixed methods feasibility study of a 12-week group outdoor health walk using activity tracker technology. Data from * Walkers or ^#^ Walk Leader.

Walker-level data came from: (i) self-report questionnaire responses (open- and closed-ended) completed at two time points (T0 and T12, Figure 1); (ii) daily step counts from walkers’ activity trackers; and (iii) post-programme interviews. Information about programme delivery came from: (i) checklists; (ii) written reflections by the Walk Leader; (iii) meeting minutes; (iv) notes from verbal discussion between the Walk Coordinator (AM) and one of the researchers (KNI); and (v) a written report.

Walkers in the 12-week intervention were informed about the research study; written consent was obtained (Figure 1). At the start of the study, participants completed the paper-based ‘entry’ questionnaire (T0), received an activity tracker and had an online account created for them by the Walk Leader. The first walk occurred one week later (T1), allowing for the collection of baseline step counts level prior to starting the activity tracker GOHW. At the final walk (T12), participants completed the paper-based ‘exit’ questionnaire. The Walk Leader kept a weekly checklist about attendance, distance walked, and the duration and type of natural environment, and noted any reflections about the experience. Data from the activity trackers were uploaded weekly to each walker’s account by the Walk Leader, who had an ‘administrator’ app which required an iPhone. 

The above detailed data collection was in addition to practices associated with the existing GOHW programme. Walk leaders keep a weekly Walk Register (e.g., recording attendance, weather conditions; T1–T12, Figure 1). New walkers have to complete a New Walker form (collected at T1, Figure 1) for national accounting purposes. These were anonymised and made available to the research team as a source of data. 

After the 12-week programme, two participants provided in-depth interviews and the Walk Leader provided a written reflection of the experience. A stakeholder meeting among national park personnel, programme delivery personnel, technology suppliers and a regional NHS representative was held. A report to the funder who supported the development, implementation and evaluation of the activity tracker GOHW was prepared by the Walk Coordinator with contribution from one of the researchers (KNI). All post-programme data were made available to the research team. 

### 2.4. Data Collection Instruments

#### 2.4.1. Study Questionnaire

This section details the study questionnaire content and the time point at which data were collected: study entry (T0) and at study exit (T12).

##### Background Information

Socio-demographic details included: age (T0; year of birth), gender (T0; write in), paid employment (T0 and T12; yes/no), ethnicity (T12; tick box list), whether they smoked (T0 and T12; yes/no) and any longstanding physical or mental condition/disability (T0; yes/no). 

A health status question assessed the presence of 15 specific health conditions (e.g., diabetes, heart disease). Participants selected all that applied in response to the question: ‘*Do you have any of the following conditions?*’ The list of included health conditions was drawn from intake forms used in existing programmes to promote physical activity. Participants could also add health conditions. The question was asked at entry (T0) only based on the assumption that there was likely to be little change in these conditions in the course of the 12 weeks. 

##### Recruitment 

A question about the main way in which participants learned about the 12-week activity tracker GOHW was included in the entry questionnaire (T0) only. Participants selected from a provided list (e.g., health professional, leaflet in letterbox) with the option to add a different response.

##### Self-report Motivators and Benefits

At entry (T0), an open-ended question asked participants to write in why they wanted to join the project. The exit questionnaire (T12) included two open-ended questions exploring factors that might motivate joining and staying involved in the activity tracker GOHW. Participants were first asked to think back to before they joined the walk and write in ‘*what was it that convinced you to try this walk*’. The second question explored ‘*what was the most important thing or things that kept you engaged’*. 

To explore their perceptions of programme benefits, participants selected from a provided list all benefits they hoped to gain (T0) and what benefits they thought that they had gained (T12). Items focused on fitness, weight, flexibility, activity level, reducing health risk, meeting people, having fun, with an option to write in benefits not listed. At programme end (T12), participants were asked to describe the main benefit gained.

##### Self-report Physical Activity

Participants were asked to indicate their main type and level of physical activity. For the first question, participants selected one type from a list: incidental (e.g., gardening or housework); regular fitness/exercise activity (e.g., walking); recreational such as swimming; organised sport; other (write in). Physical activity level was measured using the previously validated Scottish Physical Activity Screening Question (Scot-PASQ) [66], which asks participants to indicate the number of days of at least 30 min of physical activity in the previous week. For individuals who had four or fewer days, a follow-up question asked as to whether they had been physically active for at least 150 min (2.5 h) during the previous week. These were asked at both entry (T0) and exit (T12).

##### Holistic Health and Wellbeing

A modified version of the 16-item Self-Assessment of Change (mSAC) measure [54] assessed holistic biopsychosocial-spiritual health and wellbeing. The SAC tool comprises 16 visual analogue scales anchored with salient word pairs, e.g., exhausted–energized, depressed–joyful. For the mSAC scale, modifications were made to two of the word pairs to better reflect the cultural context of this study. Specifically, the word pair ‘scattered–focused’ was modified to ‘difficult to focus–focused’; ‘closed hearted–open hearted’ was altered to ‘closed to others–open to others’. Modifications were made in consultation with practitioners associated with the GOHW programme. 

Participants completed the word pair items at T0 (‘*where you believe you are right now’*) and at follow-up (T12). The follow-up stem question asked participants to reflect on ‘*life changes that you experienced since beginning the Health Walks programme*’, indicating a mark on the 100 mm blank line for both ‘*where you were before*’ and ‘*where you are now*’ for each word pair, thereby providing both a retrospective assessment of where the participant thought they were at the start of the walking programme (“Before”) along with an assessment of where they are now (“Now”). 

The SAC scale has been administered to more than 600 individuals across six different studies, including RCTs and programme evaluations [55,56]. Participants have used the full 100 mm line and have shown a willingness to indicate that domains were worsening, as well as improving. Of importance for the interpretation of results, changes of more than 10 mm on the 100 mm line are considered clinically relevant [55].

#### 2.4.2. Physical Activity Tracker

The physical activity tracker, an accelerometer (Storm Health, Edinburgh, UK) with a Bosch 3D motion sensor, was battery powered, could store up to 30 days of activity and could be worn on the wrist (like a watch) or affixed to a belt loop. Recharging the batteries required a USB charger. Activity was recorded in the form of step counts. Step count data from the activity trackers were collected electronically via the online platform and provided to the Walk Coordinator as an Excel spreadsheet at programme end. Each participant’s data were assigned a unique ID number, which served to anonymise the step count data and link across the separate anonymised data files for analysis. The step counts provided a source of objective data about physical activity.

#### 2.4.3. Walk Attendance, Distance, Duration, Natural Environs

Insight about walk attendance was drawn from the New Walker form (completed at T1 by new walkers) and the weekly Walk Register, kept by the Walk Leader. For each weekly walk, the Walk Leader additionally completed a checklist developed for the study. This provided information about the walk and the type of environment in which it took place. Walk-related information included: date of walk (write in: date); the weekly walk number (write in: the weekly walk number, e.g., 1, 2); the number of people attending (write in: number); the duration of the walk (write in: walk start time and walk end time); and the distance walked (write in: distance including source of information about distance, e.g., estimate, phone-based app). The walk environment was measured with a list of categories (e.g., natural and semi-natural places, green corridors, public spaces) that has previously been used in nature–health research [35]. The Walk Leader was asked to select the one that *‘best describes the site*’ in which the walk took place. An open-ended question asked the Walk Leader to reflect on any ‘*important observations about today’s group walk and motivation session*’. 

#### 2.4.4. Qualitative Data

Additional qualitative data came from transcripts of in-depth interviews (developed and conducted by Storm Health) with two walkers who were willing to talk about their experience, the Walk Leader’s written reflections, the Walk Coordinator’s verbal discussions, and minutes from the stakeholder meeting. The in-depth interviews focused on what aspects of the activity tracker system (e.g., tracker, apps, online account) the walker made use of and any perceived benefits from that usage. The Walk Leader was asked to write about their experience while the Walk Coordinator (AM) provided verbal reflections to one of the researchers (KNI). The stakeholder meeting focused on the experience of integrating activity trackers and their associated services into a GOHW. An additional source of qualitative data was a final project report (written by AM with contribution from KNI). 

## 3. Results

### 3.1. Participants

Thirteen individuals were recruited into the activity tracker GOHW; all thirteen volunteered to be included in this feasibility study. The average age was 71 (range 63 to 81), approximately three-quarters were women (*n* = 10, 76%), not in paid employment (76%), and nine (70%) indicated they had no long-standing physical or mental condition or disability. All were of White ethnicity. 

At programme entry (T0), the most prevalent self-report health conditions were high blood pressure, followed by heart disease. Six participants (46%) indicated more than one type of health condition (range 2 to 5). No-one smoked. One participant specifically noted that they had joined to “regain fitness after a heart attack”.

### 3.2. Recruitment-Retention-Motivation

The first objective of this feasibility study was to understand the contribution of activity trackers to the recruitment and retention of walkers. To answer this objective, data from qualitative reports from the Walk Coordinator, walker interviews and closed-ended or short answer responses from the study participants’ questionnaires were integrated.

#### 3.2.1. Recruitment to Walks and Motivation for being Involved

The Walk Coordinator provided a qualitative narrative of the recruitment process. The original implementation plan for the activity tracker GOHW included recruitment through local health centres. Initial discussions with medical doctors explored the distinction between ‘referral’ and ‘signposting’. It was agreed that ‘signposting’ would be the most appropriate way in which to position their role in terms of informing individuals about the activity tracker GOHW rather than prescribing them through a referral.

However, no one who joined the activity tracker GOHW received information through the local health centres. Ten participants learned of the programme through word-of-mouth from friends and family or others already involved in a walking group. Two were specifically asked to help with the walks and one individual learned of the walk via a leaflet through the letterbox. Four of the participants were new to a GOHW.

According to self-report questionnaires at T0, several walkers joined to improve health behaviours, such as increasing walking and maintaining weight. Others sought to improve, regain or maintain fitness and flexibility or to improve their health and wellbeing more globally. The opportunity to have fun, along with social benefits such as enjoying company or an interest in being part of the group that was forming, motivated some to join. One individual was motivated altruistically to “contribute to evaluation”. Encouragement or personal contact by the Walk Leader was a tipping point to joining for several participants. The activity tracker itself was appealing for accessing fitness data. 

#### 3.2.2. Retention and Motivation for Staying Involved

All thirteen walkers who began the programme remained involved over the 12 weeks. Qualitative short answers provided by the participants at T12 reveal that one of the most important factors in maintaining engagement with the programme was the social aspects of being part of a group, enjoying the company, or helping others. For others, getting fit and having improved mobility and other health benefits kept them engaged. Three individuals mentioned the activity tracker as a motivator for continued engagement: the weekly step counts and graphs allowed them to see their weekly progress, made them think more about their fitness level and encouraged effort to “try and do better each week”. Another motivation for staying engaged included “getting out”.

Participants, reflecting on the benefits gained after 12 weeks of the activity tracker GOHW, identified the importance of having the incentive to walk and exercise regularly, making them more fit and flexible. They valued being able to maintain their health overall; notably, one participant stated “[I] have not seen [the] doctor since I started walking” (P08), while another (P09) declared she was “happier with life” after taking part in the activity tracker GOHW. The Walk Leader noted that after about 5 weeks, some participants were less interested in the activity tracker feedback. The social interaction was an important benefit for a few, as it was “something to look forward to”.

### 3.3. Programme Implementation

The second study objective was to assess how the inclusion of activity tracker technology affected programme implementation. Implementation was influenced by the attributes and skills of the people delivering the programme, the ease of use of the activity trackers and associated online system, and how the integration of the activity tracker technology affected programme delivery. Data sources for this section include: Walk Leader written reflections, discussion with the Walk Coordinator, interviews with walkers, minutes from the stakeholder meeting, the report to funders and the experience of different users (i.e., walkers, Walk Leader and the Walk Coordinator).

#### 3.3.1. Walk Leader Characteristics

The volunteer Walk Leader was critical to the successful delivery of the activity tracker GOHW. The Walk Coordinator noted that the Walk Leader’s enthusiasm and effectiveness was essential for the support and engagement of the walkers over the 12-week intervention. In general, walk leaders receive training in techniques that form the basis of a GOHW such as: setting an appropriate walking pace; the ‘warm up, brisk walk, cool down’ stages of a GOHW; providing encouragement and motivation to continue walking; and planning appropriate walk routes through nearby natural environments. 

#### 3.3.2. Activity Tracker System

Incorporating an activity tracker system required additional training for the Walk Leader and others involved in programme delivery. This included both the technical aspects of the activity tracker and the ways in which its data could be used for motivational purposes. A range of technical issues arose during both the initial setting up of walkers with their activity trackers and the ongoing support provided during the 12-week programme. 

Trackers were considered relatively simple to use and hard-wearing, although straps and clasps were observed as not being durable. Frequent charging was required due to a short battery life. This posed difficulties for walkers who did not have access to a USB charger and an unanticipated cost to the programme as it was decided to purchase these chargers and provide them to walkers. The Walk Leader noted the potential for this to negatively impact motivation: “Walkers got ‘upset’ when their tracker failed or ran out of battery midway through the day and their steps weren’t recorded!” Additionally, both the Walk Leader and walkers noted the need for more hands-on practical training. For example, one walker (P08) noted: “My activity tracker went into sleep mode at the beginning, but I managed to get this sorted [after 2 weeks].” 

The online website was only ever accessed and utilised by the Walk Leader. This was largely because none of the walkers had access to devices (e.g., smartphones, tablets, computers) outside of the weekly walk and the syncing process could only be done using an iPhone. The Walk Leader noted that walkers were more engaged when they set a target:

*I found that walkers who wanted a target set were much more engaged throughout the 12 weeks and wanted to see their target on the computer. Those who did not want a target set were less / not interested in the weekly results by about week 5.*

However, it was not possible to amend the target on the participant’s online profile, which meant a handwritten log had to be kept in order to show whether the target was achieved or not. 

#### 3.3.3. Integration of Technology into the GOHW

GOHWs have an existing set of procedures to assure programme fidelity. The integration of the activity tracker system into such walks necessitated consideration of the processes for and impact of the use of such technology. 

##### Activity Tracker Processes

For the Walk Leader, the use of activity trackers added another dimension beyond the usual weekly management of a GOHW. The following observations from the Walk Leader highlight this with respect to the syncing process.

At the beginning, when assigning trackers to individual walkers:

*All unassigned trackers at the start of the 12 weeks needed to be charged ready for each walker—but this meant they were all picked up by the iPhone and there was no way to tell which tracker you were assigning [to which walker]. So [you] have to make sure that all but the one you physically have in your hand are out of range of the iPhone when assigning it to a walker.*

On an ongoing basis at each week’s walk:

*I found that some days it could be a bit overwhelming at the start of the walk with everyone turning up and immediately asking if they’d been synced! As we had 2 groups (slow and fast) I felt under pressure to get the slower walkers synced first as I was stopping them from going out. I walked with the faster group so was able to sync them as we walked which was much easier! Also had to keep track that I had got everyone synced and quickly check each person’s data and comment on how they had done that week and having the walk register to do!*

An additional dimension identified was related to the use of information for motivational purposes. The Walk Leader noted the “Limited time to show the graph and chat to each walker. Although only about 1/3 of the group were interested in seeing the graph”. 

##### Impact on the Walk Experience

Comments provided by the Walk Leader and walkers themselves suggested, however, that the use of an activity tracker system was a positive experience. Walkers described how the activity trackers motivated them to have a step counts physical activity target. One walker (P08) observed: “at the start of the programme my target was 1000 steps a day. My target is now 12,000 steps.” Another walker (P09) noted:

*It is a great incentive. It makes you go out and see how far you can go in a day. I’m not doing as much now as the morning and nights are darker but I did get up to 10,000 steps a day. Now I am doing between 5000–6000 steps. I did 8000 steps yesterday.*

Comments from these two walkers highlight the activity tracker as a motivation for both physical activity and getting outside, either on one’s own or with a group. An additional comment from walker P08 highlighted the motivation to get outside for a longer period of time:

*The tracker was helpful. It makes you more aware and think ‘I must go out today’, especially when it is a nice day and it makes me walk a bit further. It certainly gives me an incentive to go further.*

The activity trackers were a good motivator through social comparisons between individuals in the walking group, as illustrated in the following observations from P08: “The company in the walking groups has been excellent. We have fun teasing each other about steps. ‘I have done more than you!’ or ‘I haven’t had a good day’”. This was similarly noticed by the Walk Leader:

*[The activity tracker GOHW] gave the group a boost and a focus from the “normal” Health Walk… there was more conversations amongst the group about how much they had walked in the week, health and fitness in general...lots of excuses/reasons given by walkers as to why certain days their step count was low! But shows they are thinking about their activity.*

As evidence of walker enthusiasm, the Walk Leader noted that “everyone is still actively turning up to be synced even if they can’t make the walk” and seven of the walkers expressed an interest in continuing to use the trackers beyond the end of the 12-week programme. Walker P09 suggested that the activity tracker supported their future intentions for outdoor physical activity: “The tracker is a great incentive. I’m so glad I got it. I asked to keep it after the 12 weeks. We go (on) smaller walks round the village each week.”

### 3.4. Physical Activity and Health and Wellbeing Measures

The third objective of this study was to field-test the use of two novel measures: step counts available from activity trackers for quantification of physical activity and the mSAC scale for health and wellbeing. Data from the weekly checklist, questionnaires, interviews and the activity trackers are brought together here. 

#### 3.4.1. Walk Attendance, Distance, Duration, Natural Environs

The number of people attending any given weekly walk ranged from three to 11 walkers. Walk distance ranged from 1–1.5 miles in 30–35 min in public open spaces (e.g., village centre) and amenity green space (e.g., village green) for the ‘slow walkers’ sub-group. The ‘fast walkers’ sub-group walked primarily in farmland or natural and semi-natural places covering 1.2–2 miles in 40–45 min. 

#### 3.4.2. Physical Activity Type and Levels

The main type of activity in which participants engaged remained largely unchanged over the course of the 12-week activity tracker GOHW. Approximately two-thirds of the participants engaged primarily in ‘regular walking, running or cycling for fitness/exercise’, one third in ‘incidental activity such as housework, gardening, walking about the house or shopping’ and one person identified ‘recreational activities such as yoga/pilates/dance/swimming’. 

**Figure 2:**
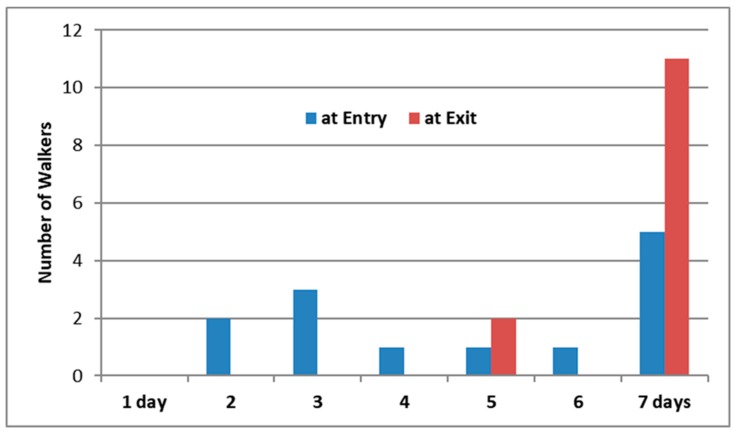
Number of physically active days in a week at entry and at the end of a 12-week activity tracker group outdoor health walk (*n* = 13 walkers). Question asked: ‘In the past week, on how many days have you been physically active for a total of 30 min or more? Physical activity may include: walking or cycling for recreation or to get to and from places; gardening; and exercise or sport which lasts for at least 10 min’.

The quantity of self-reported physical activity improved during the study (Figure 2). At the start (T0), six of the 13 participants exercised for 30 min on fewer than 5 days per week. By the end of the study, all 13 participants were physically active for at least 30 min on 5 or more days per week, thus meeting UK physical activity guidelines for both adults (age 19–64) and older adults (65+ years old) [67]. 

**Figure 3:**
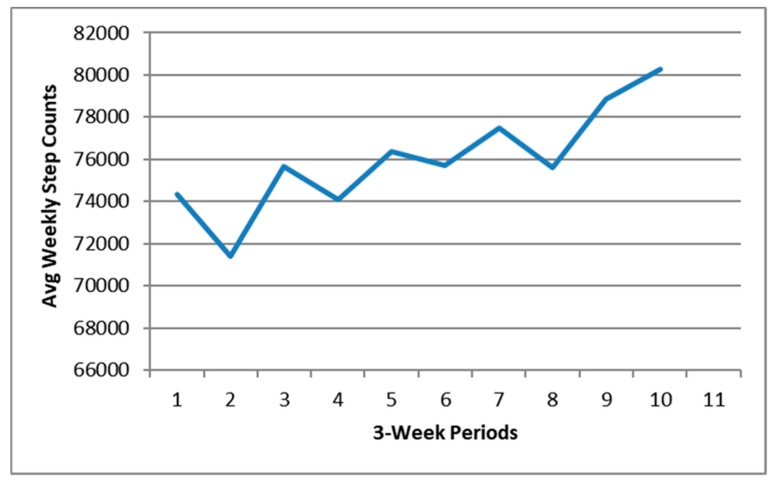
Three week moving average for the 12-week activity tracker group outdoor health walk (*n* = 13 walkers).

Activity tracker step counts allowed for objective quantification of the shift in physical activity. The average number of steps walked during the final week (*M* = 82,064; SD = 38,310) were more than at the beginning (*M* = 79,573; SD = 48,822). The simple moving average for three-week intervals demonstrates the long-term upward trend in the weekly step counts by the group as a whole (Figure 3).

#### 3.4.3. Health and Wellbeing

The mSAC measure provided an assessment of the change in participants’ perception of 16 aspects of holistic biopsychosocial-spiritual health and wellbeing, from before (T0) to after (T12) the intervention (Table 1). The mSAC was quick and easy to fill out. Data from two participants suggest that there may have been difficulty understanding the instructions: one did not provide ‘before’ assessment, the other may not have understood the difference between the ‘before’ visual analogue scale and the ‘now’ as both were marked the same for all but one item. 

Mean differences of greater than ten points are deemed clinically relevant [55]. Three physical dimensions (Sleeping, Energized, Vibrant senses), two affective or emotional aspects (Joyful, Calm), one cognitive (Focused) and one spiritual (Whole) items reached this level of change.

In qualitative interviews with two walkers, they commented on improvements in their health and wellbeing. For example, P09 said: “I feel a lot better. I feel like I have a new lease of life.” The other noted, “I am certainly sleeping better. I think going out in the fresh air helps” (P08). Both these individuals experienced more positive emotions and felt they had more energy. These identified improvements echo the findings on the mSAC scale for the whole group, supporting its face validity in this type of study. 

## 4. Discussion

This paper reports findings from a transdisciplinary feasibility study of a nature-based intervention aimed at increasing physical activity and promoting health and wellbeing. The usefulness of activity trackers as both a motivator for joining and staying involved in the programme and as a measure of physical activity was explored. The study also field-tested a holistic health and wellbeing measure from integrative medicine for use in nature-based health intervention research.

Older adult walkers were successfully recruited to, and remained involved throughout, a 12-week activity tracker group outdoor heath walk (GOHW). Numerous challenges were encountered in incorporating the activity tracker technology into the programme, however, the motivational benefits appeared to outweigh the challenges. Activity trackers allowed for the quantification of physical activity, corroborating self-report. There was a general upward trend in the weekly step counts and, by study end, all participants were meeting or exceeding UK guidelines for physical activity (30 min/day; 5+ days/wk) [67]. The holistic modified Self-Assessment of Change (mSAC) scale was an easy-to-use single measure demonstrating that walkers had changes in physical, cognitive, emotional and spiritual dimensions of health. These included: sleeping well, experiencing vibrant senses, feeling energised, focused, joyful, calm and whole.

A variety of issues were identified related to programme implementation as well as evaluative research of nature-based interventions (NBIs) that seek to promote health and wellbeing. 

### 4.1. Motivation for Joining and Maintaining Engagement 

Efforts to engage medical doctors to signpost individuals to the walk were unsuccessful. Forming effective relationships with medical practitioners required time, commitment, face-to-face engagement and local knowledge. Many other staff members in a medical practice have the opportunity to influence patients, and may have more time than the physicians, thus future efforts would benefit from the engagement of all staff members. This is consistent with a growing interest in social prescribing and use of ‘link workers’ [68,69]. For future trials, it would be beneficial to have a more proactive discussion with the practice staff to ensure that they identify a small cohort over 2–3 weeks prior to the commencement of a specific, time-limited programme. An invitation to join a meeting to hear about the programme and expectations could be issued and may aid in the recruitment of walkers.

Walkers became involved for a variety of social- and health-related reasons, driven by encouragement from others (family, friends, Walk Leader) or an intrinsic motivation to improve fitness. Study findings suggest that the anticipation of having objective data about one’s physical activity was a further reason for getting involved initially. In this group, the aspect of being out in nature was little featured among motivators to join or stay involved.

Feedback during the programme on the number of steps walked served as a motivator for individual walkers to stay involved, be more active, and increase the number of times they went outside. The step counts also generated interaction within the group, which acted as additional motivation. The results from this study are similar to those of Tocci et al. [70], who found that older adults appreciated how the tracker made them more aware of their own inactivity and motivated them to be more active. The feedback also provides a form of cognitive engagement, an element identified as a valuable contribution in other walking studies [71]. The social interaction that was generated within the group by the use of the trackers may be another way to build the kind of social connections that have been found to support continued participation [39,40]. 

### 4.2. Added Value for Programme Implementation 

The extent to which an activity tracker added value without compromising the delivery of an existing GOHW was also explored. For programme implementation, the activity tracker adds a technology component to the delivery process. The volunteer Walk Leader played a pivotal role in successful programme delivery, providing both technical support with the tracker system and motivation through review and interpretation of the change in physical activity from the activity tracker step counts. 

Findings suggest that incorporating this type of technology is possible, albeit with some caveats as its integration brought, for example, additional management responsibilities and costs, both financial (e.g., purchase of activity trackers) and time-based (e.g., lead time for sourcing technology, training for volunteers). A key lesson from these observations is the importance of having an appropriate number of support people available to implement all procedures and processes effectively and efficiently.

The use of technology within a GOHW raises relevant questions about anonymity, confidentiality and ethics particularly when delivering such a programme within a research context. Navigating data-sharing agreement requirements, and associated ethical permissions was more time-consuming and complex than planned for by the delivery organisation. The extent of the consent required from walk participants was daunting for some and, although not a barrier for this feasibility study, could be one amongst other walkers. Both additional time and preparation for walkers and volunteers are needed for successful implementation.

### 4.3. Measurement

This study provides insight into the feasibility of using step counts as an intermediate measure and the mSAC scale as an outcome measure in field studies of nature-based interventions.

#### 4.3.1. Intermediate Measure: Physical Activity

The activity trackers were useful as a measure for physical activity, which included both the activity from taking part in the weekly activity tracker GOHW and the physical activity level in ordinary daily living, in the form of daily step counts. This quantitative measure is a valuable improvement, for research purposes, when compared to self-report of physical activity [72,73], previously used in GOHWs [33,37]. There are, however, several issues to be considered. First, accuracy can vary depending on walk setting and where the tracker is worn. Activity trackers are more accurate in limited settings such as treadmill exercise than in over-ground walking or free-living [74], and when worn on the hip or upper torso rather than the wrist [75]. Yet, the majority of people prefer to wear trackers on their wrists [76] and, in this study, activity was over-ground and free-living, thus introducing an element of error into the measurement. Our assumption is that the error would be relatively consistent across the time span and conditions of the study. If reasonably accurate, this quantification could be useful in providing a continuous variable to use in regressions. Second, these devices typically appeal more to males and to younger people [76]. The fact that this group of older adults, the majority of whom were women, stuck with using the activity trackers indicates that this is a promising form of data collection for an important mediator of the effect of nature-based activity on health or as a short-term outcome.

#### 4.3.2. Downstream Health and Wellbeing Outcome: Holistic Health and Wellbeing

This study field-tested the use of the mSAC scale as a parsimonious way to measure holistic health and wellbeing outcomes that may come from interaction with natural environments. The domains covered in the mSAC scale encapsulate the broad spectrum of benefits endorsed in previous qualitative interviews with park users [41] and highlighted in the work of others [23]. 

The minor modification of the recently developed SAC measure for assessing complex interventions was easy for participants and researchers to use. The 16-item scale is less burdensome than administering multiple validated scales to cover all domains, as has been done in other studies [77]. As a measure, it is sensitive to change and can provide clinically relevant data. The changes measured in this study using the mSAC scale matched well with the qualitative findings identified in the two walkers’ interviews, supporting its face validity for NBIs. The changes in sleep quality identified here add to the growing list of potential health benefits from nature to investigate further. 

### 4.4. Activity Tracker Group Outdoor Health Walks 

Although this was a feasibility study, findings support that a 12-week combined activity tracker and GOHW programme can promote increased physical activity as well as improved holistic health and wellbeing. As suggested by the mixed methods approach, monitoring, goal setting, encouragement from the Walk Leader and social support were important factors which is consistent with findings in the activity tracker field [43]. By programme end, all 13 participants were meeting national physical activity standards and endorsed clinically relevant changes in multiple dimensions of health and wellbeing. These findings are consistent with previous outcomes from the GOHW literature, including increased physical activity [19], mental wellbeing [33,35] and positive affect [31,32] along with reduced depression [28], perceived stress [30], and negative affect [30,34]. The findings of sleeping well, feeling energised, and feeling wholes may warrant further investigation as they have not been explored as thoroughly as other noted outcomes. 

### 4.5. Study Limitations 

Feasibility studies have several inherent limitations, including small sample size, limited socio-demographic diversity, and lack of a control condition, issues which are of relevance for the study reported here. Study participants were of a limited age range and ethnic diversity; the majority were female. This demographic is, however, similar to other UK studies of GOHWs [33]. The study took the form of a pretest–posttest evaluation rather than a controlled trial, so it may be that physical activity and health and wellbeing would change as much with just an ordinary delivery of a GOHW (i.e., without an activity tracker). Given the small sample, one can anticipate wide variability, therefore reporting results from inferential statistical analyses is not appropriate or particularly useful. However, means and standard deviations are provided for utility in planning future sample sizes, as is recommended for the reporting of feasibility studies [57]. Additionally, by incorporating complementary qualitative data, this study provides a greater depth of understanding from a range of viewpoints to address the feasibility study objectives [61]. 

### 4.6. Future Research 

**Figure 4:**
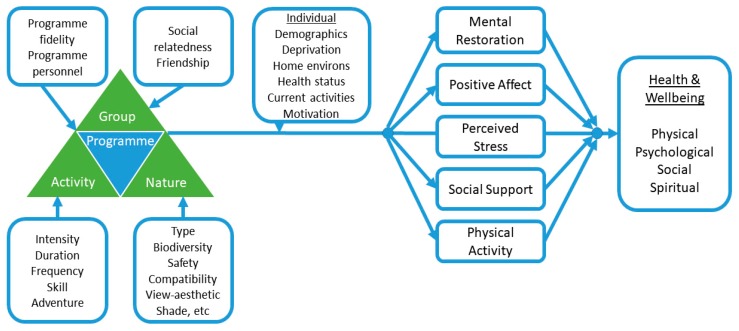
Conceptual model for investigating nature-based interventions that aim to promote health and wellbeing through behaviour change. The model illustrates components of the intervention, possible moderators, mediators and downstream health and wellbeing outcomes. Drawn from [23,37,41,82,83,84].

Based on insight from this feasibility study, future NBI studies of GOHWs (with or without activity trackers) should consider conceptualising GOHWs as a complex intervention [78] requiring innovative methods of study and appropriate measures. To guide evidence generation that will influence health practitioners, public health officials and health funders, a clear conceptual model is useful [79]. This will promote investigations of the direct and mediated effects of the components of this complex intervention on meaningful health outcomes. Figure 4 provides a conceptual model that illustrates the proposed components of a GOHW type NBI and identifies key concepts to measure. The complex intervention of a GOHW/NBI consists of four components: the activity of walking, the influence of nature, the effects of being part of a group, and programme delivery effects. The model additionally details potential mediating pathways through which such NBIs may affect downstream health and wellbeing. The health and wellbeing outcomes are specified according to the biopsychosocial–spiritual model of health, as has previously been suggested [41]. The model also incorporates attributes of the individual and their life context that potentially modify the associations between NBIs and health and wellbeing outcomes. This conceptual model draws on multiple health disciplines (public health, medicine, sports medicine), two key restorative environment theories from environmental psychology [80,81], elements from four published models [23,82,83,84], and research by the authors [37,41].

Future studies could benefit from using a complex intervention lens to frame and understand how NBIs such as GOHWs may promote health. Such studies could address multiple aspects of the proposed model. In a larger study, following this model, statistical regression and mediation analysis could elucidate the relationship of the complex intervention, physical activity, and holistic health and wellbeing that is suggested by our feasibility data.

### 4.7. Implications

Although this is a feasibility study, it provides justification for further research that would support the local, regional and national initiatives that focus on prevention, early intervention and self-management. For example, within the UK, the NHS, Scottish Government [8] and local governments [85] advocate for the use of community-led group walks in natural environments as a social prescribing intervention for increasing physical activity and positive mental health. The use of activity trackers with these community-wide approaches may be a motivator for joining. This study suggests the importance of building bridges locally between health care professionals and GOHW volunteers/professionals to facilitate the recruitment of inactive people to GOHWs. There is also an investment needed in technology (e.g., activity trackers), infrastructure to support volunteer walk leaders (e.g., motivational training), and recruitment of these dedicated volunteers. There are challenges to address when embedding evaluative research into existing ‘real-world’ programmes. These challenges are mitigated by the fruitfulness of working collaboratively across sectors among academic researchers, practitioners delivering the programme, and healthcare professionals promoting health. The findings from this feasibility study suggest that future prospective, controlled studies with larger sample sizes could produce the type of evidence needed to support the contributions of activity tracker GOHWs to physical activity and resultant holistic health and wellbeing promotion.

## 5. Conclusions

The 12-week, nature-based intervention, an activity tracker group outdoor health walk, facilitated thirteen individuals to increase their physical activity and experience positive changes in several dimensions of health and wellbeing. The incorporation of activity trackers enhanced the recruitment of older adults to the intervention. The activity tracker feedback aided in motivation and retention, particularly for the previously sedentary individuals who were new to the programme. This study added a technology component to the programme delivery of a complex intervention in a natural environment. Successful integration of the activity tracker technology can be aided by: trackers that are available, durable and simple to use; committed volunteer walk leaders; the provision of appropriate training to walkers and walk leaders; and carefully coordinated effort and communication across sectors. The activity trackers allowed for the quantification of an important hypothesized mediator of nature-based interventions effects on health, i.e., physical activity. The modified Self-Assessment of Change scale identified changes in health and wellbeing that correspond with both theory [80,81] and the biopsychosocial–spiritual model [41]. The identified effect of group outdoor health walks in nature on sleep has been minimally investigated. These are promising findings given a recent report on the barriers associated with the usage of the outdoors by older people [64]. Amid continued calls for evidence on the effectiveness of nature-based interventions [22], a conceptual model is provided for future research into complex nature-based interventions, such as group outdoor health walks, that aspire to promote health through behavioural change.

## Figures and Tables

**Table 1:** Mean and standard deviation for health and wellbeing measured at T12 by the modified Self-Assessment of Change (mSAC) scale comprised of sixteen 100 mm visual analogue scales (VAS) anchored with salient word pairs (*n* = 12 walkers).

Health and Wellbeing VAS Anchoring Word Pairs		Before Mean (SD)	Now Mean (SD)
Not sleeping well	Sleeping well	55.25 (30.82)	68.08 (30.21) ^1^
Exhausted	Energised	54.50 (25.00)	71.67 (27.13) ^1^
Dull senses	Vibrant senses	61.45 (23.26)	73.82 (21.96) ^1^
Body does not recover quickly	Body recovers quickly	76.00 (23.23)	80.00 (22.85)
Scattered	Focused	63.17 (30.35)	74.17 (27.13) ^1^
Stuck	Letting go	64.58 (28.19)	68.83 (29.39)
Defined by my illness or problems	Not defined by my illness or problems	65.25 (29.46)	69.50 (31.04)
Hopeless	Hopeful	70.67 (23.62)	79.08 (15.84)
Depressed	Joyful	68.25 (26.34)	78.50 (21.85) ^1^
Anxious	Calm	62.25 (30.51)	72.58 (26.53) ^1^
Closed to others	Open to others	77.50 (17.26)	81.17 (16.35)
Isolated	Connected	68.92 (27.21)	77.58 (22.35)
Blaming	Forgiving	70.75 (18.90)	74.08 (19.15)
Overwhelmed	Empowered	64.25 (24.80)	72.67 (19.96)
Broken	Whole	70.50 (26.88)	81.25 (18.90) ^1^
Unbalanced	Balanced	71.92 (24.96)	79.58 (19.34)

^1^ Mean difference between retrospective ‘Before’ the programme and ‘Now’ is 10 or more points suggesting clinical relevance [55].
